# Uncovering the diversity in the *Amazophrynella minuta* complex: integrative taxonomy reveals a new species of *Amazophrynella* (Anura, Bufonidae) from southern Peru

**DOI:** 10.3897/zookeys.563.6084

**Published:** 2016-02-15

**Authors:** Rommel R. Rojas, Juan C. Chaparro, Vinícius Tadeu De Carvalho, Robson W. Ávila, Izeni Pires Farias, Tomas Hrbek, Marcelo Gordo

**Affiliations:** 1 Programa de Pós-graduação em Genética Conservação e Biologia Evolutiva- Instituto Nacional de Pesquisas da Amazônia-INPA, Av. André Araújo, 2936, Manaus, Brazil; 2 Laboratório de Genética e Evolução Animal, Departamento de Genética, ICB, Universidade Federal do Amazonas, Av. Gen. Rodrigo Octávio Jordão Ramos, 3000, Manaus, Brazil; 3 Museo de Historia Natural, Universidad Nacional de San Antonio Abad del Cusco, Peru; 4 Programa de Pós-Graduação em Biodiversidade e Biotecnologia, Av. Gen. Rodrigo Octávio Jordão Ramos, 3000, Manaus, Brazil; 5 Universidade Regional do Cariri, Centro de Ciências Biológicas e da Saúde, Departamento de Ciências Biológicas, Campus do Pimenta, Rua Cel. Antônio Luiz, 1161, Bairro do Pimenta, Crato, Brazil; 6 Departamento de Biologia, ICB, Universidade Federal do Amazonas, Av. Gen. Rodrigo Octávio Jordão Ramos, 3000, Manaus, Brazil

**Keywords:** Amphibian, Tree Toad, conservation, Southern Peru, integrative taxonomy

## Abstract

A new species of the genus *Amazophrynella* (Anura, Bufonidae) is described from the departments of Madre de Dios, Cusco and Junin in Peru. An integrative taxonomy approach is used. A morphological diagnosis, morphometrics comparisons, description of the advertisement call, and the phylogenetic relationships of the new species are provided. *Amazophrynella javierbustamantei* **sp. n.** differs from other species of *Amazophrynella* by: intermediate body-size (snout-vent length 14.9 mm in males, n = 26 and 19.6 mm in females, n = 20), tuberculate skin texture of body, greatest hand length of the *Amazophrynella* spp. (3.6 mm in males, n = 26 and 4.6 mm in females, n = 20), venter coloration yellowish, tiny rounded black points covering the venter, and thirteen molecular autapomorphies in the 16S RNA gene. Its distribution varies from 215 to 708 m a.s.l. This discovery highlights the importance of the remnant forest in preserving the biodiversity in Peru, and increase in seven the species formally described in the genus *Amazophrynella*.

## Abstract (es)

Describimos una nueva especie del género *Amazophrynella* (Anura, Bufonidae) del Perú de los Departamentos de Madre de Dios, Cusco y Junin de Peru. Utilizamos un método de taxonomía integrativa. Obtuvimos la diagnosis morfológica, comparaciones morfométricas, descripción del canto de reproducción y las relaciones filogenéticas de la nueva especie. *Amazophrynella javierbustamantei* **sp. n.** difiere de las otras *Amazophrynella* spp. por poseer tamaño medio (Hocico-cloaca en machos 16.9 mm, n = 26 y en hembras 19.6 mm, n = 20); textura de la piel tuberculada; tamaños de las manos mayores (3.6 mm en machos, n = 26 y 4.6 mm en hembras, n = 20); coloración ventral amarillento-pálida, pequeños puntos redondos de color negro en el vientre y por trece autopomorfias moleculares en el gen 16S RNA. Su distribución varía desde 215 m hasta 708 m a.s.n.m. Este descubrimiento resalta la importancia de los remanentes de la selva Peruana en términos de conservación, e incrementa en siete las especies formalmente descritas en del género *Amazophrynella*.

## Abstract (pt)

Descrevemos uma nova espécie do gênero *Amazophrynella* (Anura, Bufonidae) dos departamentos de Madre de Dios, Cusco e Junin do Peru. Utilizamos um método de taxonomia integrativa. Apresentamos a diagnose morfológica, comparações morfométricas, descrevemos o canto de anúncio e geramos uma hipótese filogenética da nova espécie. *Amazophrynella javierbustamantei* **sp. n.** difere das outras *Amazophrynella* spp. por possuir tamanho médio (Comprimento rostro-cloacal 16.9 mm em machos, n = 26 e 19.6 mm em fêmeas, n=20); textura da pele tuberculada; tamanhos das mãos maiores (3.6 mm em machos, n = 26 e 4.6 mm em fêmeas, n = 20); coloração ventral amarelo-clara, coberta por pequenos pontos redondos pretos e por treze autapomorfias moleculares no gene 16S RNA. Sua distribuição varia entre os 215 m até os 708 m a.n.m. Nossa descoberta aumenta a importância dos remanescentes da floresta Peruana em termos de conservação e incrementa em sete as espécies formalmente descritas no gênero *Amazophrynella*.

## Introduction

Until 2012, two species of *Amazophrynella* were placed in the genus *Dendrophryniscus* Jimenez de la Espada, 1868. Fouquet et al. (2012a) recognized that species of *Dendrophryniscus* from the Amazon and Atlantic rainforests represented morphologically and genetically deeply divergent lineages, and thus the authors proposed a new genus: *Amazophrynella* Fouquet, Recorder, Texeira, Cassimiro, Amaro, Camacho, Damaceno, Carnaval, Moritz & Rodrigues, 2012 for the Amazonian species *Amazophrynella minuta* and *Amazophrynella bokermanni*.

In the following years, an additional four new species of the genus were described: *Amazophrynella vote* Ávila, Carvalho, Gordo, Ribeiro & Morais, 2012 and *Amazophrynella manaos* Rojas, Carvalho, Gordo, Ávila, Farias & Hrbek, 2014 based on morphology; *Amazophrynella amazonicola* and *Amazophrynella matses* Rojas, Carvalho, Gordo, Ávila, Farias & Hrbek, 2015, based on morphology and genetic data (Ávila et al. 2012; Rojas et al. 2014, 2015). Species of the genus are distributed in nine South American countries: Bolivia, Peru, Ecuador, Colombia, Venezuela, Guiana, French Guiana Brazil, and presumably in Suriname (Frost et al. 2015).

Using a phylogenetic analysis based on mitochondrial and nuclear genes (Fouquet et al. 2007, 2012a), the existence of three independent evolutionary lineages was discovered within the nominal species *Amazophrynella minuta* from Ecuador and French Guianas; subsequently, other independent evolutionary lineages were discovered from Brazil and Peru (Rojas et al. 2014, 2015). The difficulties in delimiting species within the *Amazophrynella minuta* species complex resides in the relatively generalized diagnosis (see Melin 1941) and the poor geographic sampling. For these reasons, historically, the name *Amazophrynella minuta* has been used for individuals distributed throughout the Amazonian biome (e.g. Duellman 1978; Zimmerman and Rodrigues 1990; Magnusson and Hero 1991; Rodrigues and Duellman 1993; Duellman and Mendelson 1995; Fouquet et al. 2012a). Thus, taxonomy and systematics of populations that are currently part of the *Amazophrynella minuta* complex remains largely unresolved (Rojas et al. 2014), in turn limiting the knowledge of the true taxonomic diversity of the genus (Ávila et al. 2012; Rojas et al. 2014, 2015).

Given this scenario, herein is described an additional new species of *Amazophrynella* from the departments of Madre de Dios, Cusco and Junin, Peru, founded on the principles of integrative taxonomy. Morphological, morphometric, bioacoustic and phylogenetic relationships are provided as evidence for the existence of the new taxon.

## Material and methods

### Morphology

Forty eight specimens previously identified as *Amazophrynella minuta* (Melin, 1941), deposited at the Museo de Historia Natural del Cusco, Universidad Nacional de San Antonio Abad del Cusco (MHNC) and Museo de Historia Natural de la Universidad Nacional Mayor de San Marcos (MHNSM) were analyzed. This material was compared with twenty preserved specimens of *Amazophrynella minuta* from the type locality (Taracuá mission, on the right bank of the Uaupés River, municipality of São Gabriel da Cachoeira, Brazil), deposited in the Collection of Amphibians and Reptiles of the Instituto Nacional de Pesquisas da Amazônia–INPA, Brazil (INPA-H). Further comparisons were made with three syntypes deposited at the Naturhistoriska Museet, Göteborg, Sweden (NHMG), and the original description of the species (Melin 1941).

Additionally five preserved specimens of *Amazophrynella bokermanni* (Izecksohn, 1993) from near the type locality (Juruti, 30 Km from type locality), the holotype and paratypes of *Amazophrynella manaos* deposited in the Collection of Amphibians and Reptiles of the Instituto Nacional de Pesquisas da Amazônia–INPA, Manaus, Amazonas, Brazil (INPA-H), the holotype of *Amazophrynella vote*, deposited in the Coleção Zoológica de Vertebrados of the Universidade Federal de Mato Grosso–UFMT, Cuiabá, Mato Grosso, Brazil (UFMT-A), seventeen paratypes deposited in the Collection of Amphibians and Reptiles of the Instituto Nacional de Pesquisas da Amazônia–INPA, Manaus, Amazonas, Brazil (INPA-H), and the holotype and paratypes of *Amazophrynella amazonicola* and *Amazophrynella matses*, deposited at the Museo de Zoologia de la Universidad Nacional de la Amazonia Peruana (MZUNAP) were analyzed (see Appendix 1 listing all the revised specimens).

Morphological character analyses were carried out according to Cruz and Fussinato (2008) and Fouquet et al. (2012a). Sex was determined by gonad analysis.

Measurements were carried out with a digital caliper following Kok and Kalamandeen (2008) and Duellman (1978). SVL (snout-vent length) from the tip of the snout to the posterior edge of the cloaca; HL (head length) from the posterior edge of the jaw to the tip of the snout; HW (head width), the greatest width of the head, usually at the level of the posterior edges of the tympanum; ED (eye diameter); IND (internarinal distance), the distance between the edges of the nares; SL (snout length) from the anterior edge of the eye to the tip of the snout; HAL (hand length) from the proximal edge of the palmar tubercle to the tip of Finger III; UAL (upper arm length) from the edge of the body insertion to the tip of the elbow; THL (thigh length) from the vent to the posterior edge of the knee; TL (tibia length) from the outer edge of the knee to the tip of the heel; TAL (tarsal length) from the heel to the proximal edge of the inner metatarsal tubercle; FL (foot length) from the proximal edge of the inner metatarsal tubercle to the tip of Toe IV. Diagnosis of characters follow Chaparro et al. 2015.

*Statistical analysis*. We used a total of 80 adult males of the *Amazophrynella minuta* species complex (numbers of individuals and populations of origin in parentheses): *Amazophrynella minuta* sensu stricto (n = 23, from Taracuá), *Amazophrynella amazonicola* (n = 15, from Puerto Almendras and Fazenda Zamora); *Amazophrynella matses* (n = 13, from Nuevo Salvador) and the new species of *Amazophrynella* (n = 29, from Tambopata, Nuevo Arequipa, Candamo and Inambari).

All morphometric measures were log10 transformed to conform to requirements of normality (Hayek et al. 2001). The effect of size was removed from all variables by regressing them against SVL and using the residuals of each variable in a Principal Component Analysis (PCA). Significance of morphometric differences was tested with Multivariate Analysis of Variance (MANOVA) with the two first principal components being treated as dependent variables and species as independent variables. The first two principal components were used since they explained the majority of observed variation in shape. A Discriminant Function Analysis (DFA) was performed to test classification of individuals in predicted groups. All the statistical analysis were performed in R (R Development Core Team 2011) adopting a 5% significance cut-off. PCA was used to detect groups representing putative cryptic species and DFA was subsequently applied to identify the set of characters that best diagnose those groups (Padial and De la Riva 2009). Additionally we noted large size in the HAL of the new species of *Amazophrynella*, and we used an Analysis of Variance (ANOVA) of the original data (from *Amazophrynella minuta*, *Amazophrynella matses*, *Amazophrynella amazonicola* and the new species) to statistically support this hypothesis.

### Molecular data

*Laboratory procedure.* Total DNA was extracted from muscle tissue using standard phenol/chloroform extraction (Sambrook et al. 1989). A 480 bp fragment of the 16S rDNA was PCR amplified using the 16Sar and 16Sbr primers (Palumbi 1996). Amplification was carried out under the following conditions: 60 s hot start at 92 °C followed by 35 cycles of 92 °C (60 sec), 50 °C (50 sec) and 72 °C (1.5 min). Final volume of the PCR reaction was 12 μl and contained 4.4 μL ddH_2_O, 1.5 μL of 25 mM MgCl_2_, 1.25 μL of 10 mM dNTPs (2.5mM each dNTP), 1.25 μL of 10x buffer (75 mM Tris HCl, 50 mM KCl, 20 mM (NH_4_)_2_SO_4_), 1 μL of each 2 μM primer, 0.3 μL of 5 U/μL DNA Taq Polymerase (Biotools, Spain) and 1 μL of DNA (about 30 ng/μL). Sequencing reactions were carried out according to the manufacturer’s recommendation for the ABI BigDye Terminator cycle sequencing mix, using 16Sa primer and an annealing temperature of 50 °C. Sequencing reactions were precipitated using standard EDTA/EtOH protocol, and resolved in an ABI 3130xl automatic sequencer.

*Phylogenetic analysis.* We obtained 16S rDNA sequence data from two specimens of the new species (Accession numbers: KR905184, KR905185), two paratypes of *Amazophrynella vote* (Accession numbers: KF433970, KF433971), two specimens of *Amazophrynella bokermanni* (Accession numbers: KF433975, KF433976), two topotypic specimens of *Amazophrynella minuta* (Accession numbers: KF792834, KF792836), two paratopotypes of *Amazophrynella matses* (Accession number: KP681688, KP681689), the holotype and one paratopotype of *Amazophrynella amazonicola* (Accession number: KP681868, KP681669) and two paratypes of *Amazophrynella manaos* (Accession number: KF433954, KF433957) deposited in the tissue collection of the Laboratório de Evolução e Genética Animal of the Universidade Federal do Amazonas (CTGA-ICB/UFAM). The dataset also included two sequences of Amazophrynella sp. aff. minuta (Accession number: AY326000, DQ158420) from Darst and Canatella (2004), Pramuk (2006) and two sequences of Amazophrynella sp. aff. manaos (Accession number: EU201057, JN867570) from Fouquet et al. (2007). As outgroups we used species of the sister taxon *Dendrophryniscus* (see Table 2 for samples information).

Sequences were aligned using the Clustal W algorithm (Thompson et al. 1996) implemented in BioEdit (Hall 1999) and alignment was adjusted as necessary against the secondary structure of the 16S rDNA. The existence of lineages in a phylogenetic tree-based context (Baum and Donoghue 1995) was performed using Maximum Likelihood analysis (Felsenstein 1981) in the program Treefinder (Jobb 2008) using the GTR+I+G model of substitution, selected via Akaike information criterion as implemented in Modeltest 3.7 (Posada 2006). Phylogenetic support was assessed via 10 000 non-parametric bootstrap (Felsenstein 1985). Additionally uncorrected pairwise genetic distances between linages identified by phylogenetic inference of *Amazophrynella* were calculated in MEGA 5.05 (Tamura et al. 2007).

*Molecular species delimitation.* Evolutionary lineages are diagnosed by discontinuities in character variation among lineages, and correspond to phylogenetic species. The existence of lineages is therefore a necessary and sufficient prerequisite for inferring the existence of a species under the different conceptualizations of the Phylogenetic Species Concept (PSC) (Cracraft 1983; Baum and Donoghue 1995; De Queiroz 2007). The existence of lineages in a non-tree-based context (Cracraft 1983) was inferred using Population Aggregation Analysis performed at the level of an individual (Davis and Nixon 1992; Rach et al. 2008) using the dataset with the *Amazophrynella minuta* species complex: *Amazophrynella matses*, *Amazophrynella minuta*, *Amazophrynella amazonicola* and the new species. The analyses were performed in the program R (R Development Core Team 2011).

### Bioacoustics

We analyzed one advertisement call obtained from the CD of Frogs of Tambopata, Peru (Macauly Library of Natural Songs and Cornell Laboratory of Ornithology) by the authors Cocroft et al. (2001) from the Natural Reserve of Tambopata, a locality of occurrence of the new species. The call was edit with the software Audacity 1.2.2 for Windows (Free Software Foundation Inc. 1991). The spectral and temporal parameters of the recording were analyzed in the software Raven Pro. 1.3 for Windows (Cornell Laboratory of Ornithology). The advertisement call was obtained from one male in a temperature 25 °C (Crocoft et al. 2001). We measured the following quantitative parameters: call duration (seconds); pulses per call; length of silence between calls (seconds); dominant frequency (kHz); fundamental frequency (kHz) and time to peak at maximum frequency (seconds).

## Results

### Phylogenetic analysis and systematics

**Figure 1.**
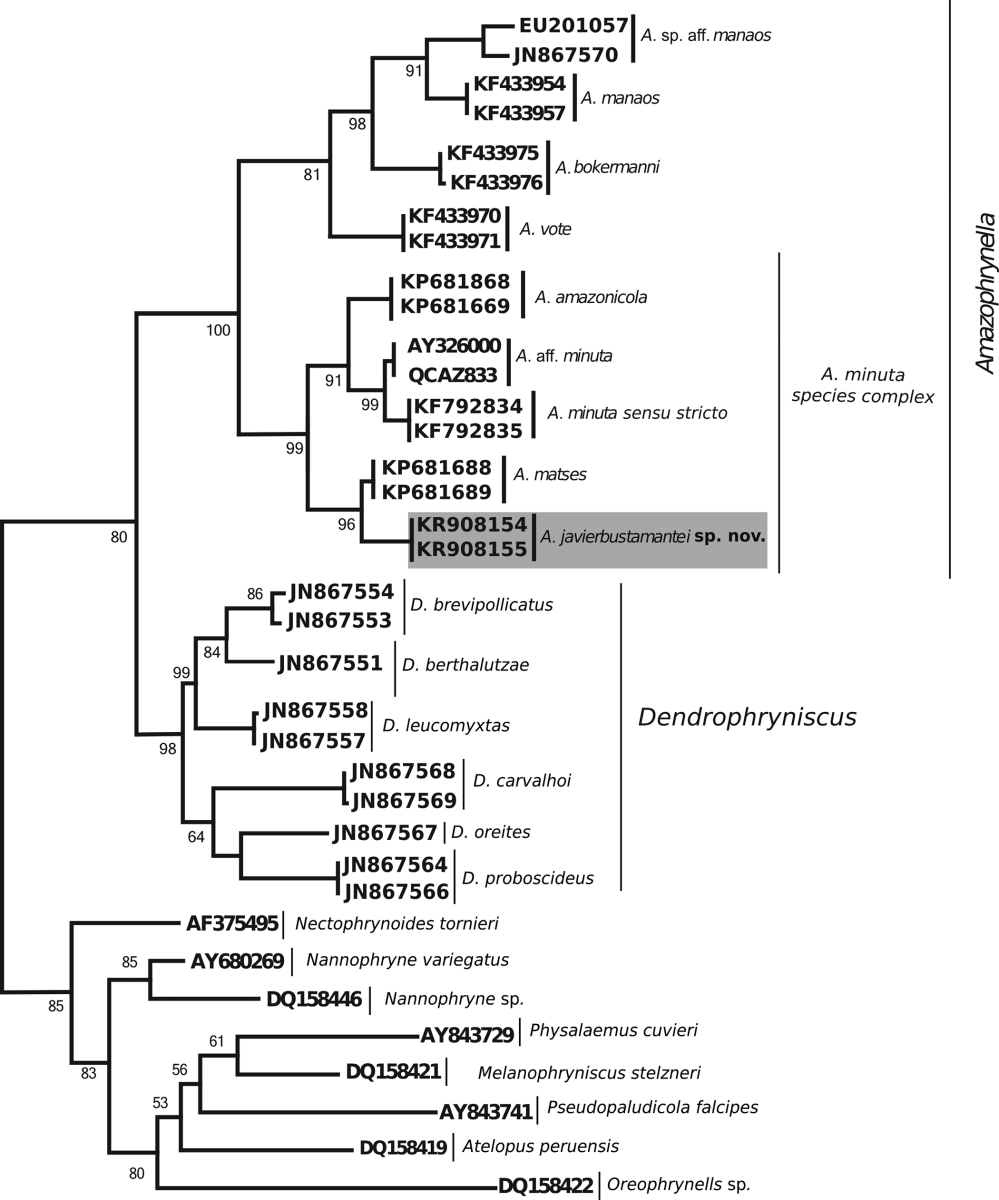
Maximum Likelihood tree of the *Amazophrynella* species based on the GTR+I+G model, analyzing 480 bp of 16S rDNA. Numbers below branches represent bootstrap support for 10 000 pseudoreplications.

In the resulting phylogeny, the six nominal species of *Amazophrynella* were recognized as monophyletic (Fig. 1). In the genus we can distinguish two monophyletic groups: One clade (bootstrap support = 100) formed by the species: *Amazophrynella manaos*, *Amazophrynella bokermanni* and *Amazophrynella vote* and another represented by the species of the *Amazophrynella minuta* “species complex” (bootstrap support = 98): *Amazophrynella minuta*, *Amazophrynella amazonicola*, *Amazophrynella matses* and the new species described herein.

In the first clade the *Amazophrynella* species: *Amazophrynella manaos* is sister taxon of the possible new specie from the Guiana Shield: Amazophrynella sp. aff. manaos (bootstrap support= 91), and both are sister to *Amazophrynella bokermanni* (bootstrap support= 98). *Amazophrynella vote* is sister of *Amazophrynella bokermanni* + (*Amazophrynella manaos* + Amazophrynella sp. aff. manaos) with a bootstrap support of 81.

The second clade corresponding to the *Amazophrynella minuta* “species complex”, *Amazophrynella amazonicola* is sister of *Amazophrynella minuta* + Amazophrynella sp. aff. minuta from western Amazonia (bootstrap support= 99). Our analysis further highlighted the occurrence of a new monophyletic lineage (*Amazophrynella javierbustamantei* sp. n.) showing sister relationship with *Amazophrynella matses* (bootstrap support = 96), both being in turn sister group of *Amazophrynella amazonicola* + (*Amazophrynella minuta* + Amazophrynella sp. aff. minuta) with a bootstrap support of 99.

Smallest uncorrected 16S rDNA p-distances estimated between phylogenetic linages was observed between *Amazophrynella minuta* and Amazophrynella sp. aff. minuta (= 3%). Greatest interspecific distance (= 14%) was observed between *Amazophrynella javierbustamantei* sp. n. and *Amazophrynella bokermanni* and was comparable to divergence observed between *Amazophrynella manaos* and *Amazophrynella minuta*. Within the “*Amazophrynella minuta*” species complex, the new species shows a high degree of genetic divergence from *Amazophrynella minuta* (= 7%), *Amazophrynella amazonicola* (= 9%) and minor genetic distance with their sister taxon *Amazophrynella matses* (= 3%) (see all pairwise genetic distance values summarized in Table 3). According to the Population Aggregation Analysis, the newly identified lineage was also diagnosable by thirteen molecular autapomorphic characters (Table 4) leading us to the conclusion that this lineage corresponds to a new species.

### Morphometric analysis

**Figure 2.**
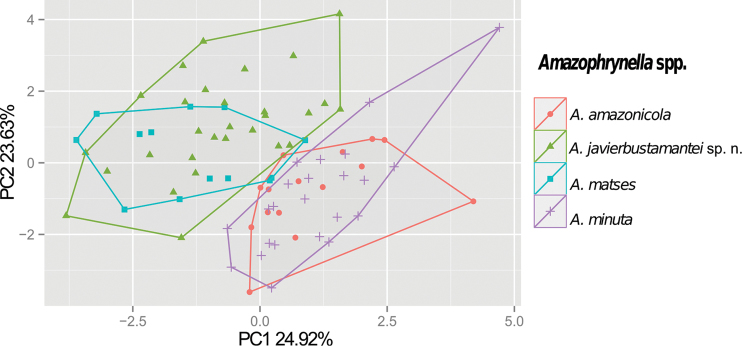
Principal Component Analysis (PCA) from: *Amazophrynella minuta* species complex. See Table 5 for character loadings on each component.

Comparative analysis of quantitative morphological data allowed us to distinguish *Amazophrynella* sp. n. from the other members of the *Amazophrynella minuta* “species complex”. The first two principal components extracted by the PCA account for 48.56% of the variation found in the dataset. The first component (PC1) explained 24.93% of total variation. In the first principal component axis, *Amazophrynella amazonicola* is distinguished from the other species due to its larger size (SVL = 14.9 ± 0.7 mm, see Table 1), sharing relative size with *Amazophrynella minuta* sensu stricto (SVL = 13.5 ± 0.6 mm, see Table 1), the species *Amazophrynella matses* is distinguish by having the smallest size of the genus (SVL range= 12.1 ±0.6 mm, see Table 1), and shares this characteristic with *Amazophrynella* sp. n. (SVL = 14.9 ± 0.9 mm, see Table 1) (Fig. 2). The second component explains 23.63% of the variation. This axis represents a shape variation vector; in this axis *Amazophrynella javierbustamantei* sp. n. is well distinguished from the three formally described species, sharing more similarity with *Amazophrynella matses* (Table 5).

**Table 1.** Measurements (mm) of adult male specimens (including the holotype) in the type series *Amazophrynella* spp. Mean ± standard deviation, with ranges in parentheses. Abbreviations are defined in material and methods.

Variable	*Amazophrynella minuta* sensu stricto (n = 15)	*Amazophrynella manaos* (n = 29)	*Amazophrynella bokermanni* (n = 5)	*Amazophrynella vote* (n = 14)	*Amazophrynella amazonicola* (n = 15)	*Amazophrynella matses* (n = 13)	*Amazophrynella javierbustamantei* sp. n. (n = 26)
**SVL**	13.5 ± 0.6 (12.5–14.2)	14.2 ± 0.7 (12.3–15.0)	16.8 ± 1.4 (14.6–18.2)	13.1 ± 0.7 (12.0–14.1)	14.5 ± 0.7 (13.3–15.4)	12.1±0.6 (11.5–13.5)	14.9 ± 0.9 (12.7–16.4)
**HW**	4.2 ± 0.2 (4.0–4.3)	4.2 ± 0.3 (3.7–4.7)	3.2 ± 0.3 (2.5–3.3)	4.0 ± 0.7 (3.3–4.4)	4.4 ± 0.3 (4.2–4.6)	3.6 ±0.2 (3.1–3.8)	4.2 ± 0.2 (3.5–4.7)
**HL**	4.9 ± 0.2 (4.8–5.3)	5.3 ± 0.3 (4.7–5.6)	3.4 ± 0.4 (2.8–3.8)	4.6 ± 0.3 (4.0–5.2)	5.2 ± 0.3 (5.0–6.2)	4.3 ± 0.3 (3.9–4.8)	5.1 ±0.3 (4.4–5.6)
**SL**	2.3 ± 0.1 (2.2–2.5)	2.7 ± 0.2 (2.3–2.7)	3.0 ± 0.4 (2.2–3.1)	2.1 ± 0.2 (1.9–2.6)	2.4 ± 0.2 (2.2–2.5)	2.0 ± 0.3 (1.6–2.3)	2.2 ± 0.2 (1.7–2.6)
**ED**	1.4 ± 0.1 (1.3–1.5)	1.3 ± 0.1 (1.2–1.6)	1.8 ± 0.2 (1.5–2.0)	1.3 ± 0.1 (1.2–1.5)	1.2 ± 0.1 (0.9–1.2)	1.1 ± 0.1 (0.9–1.2)	1.3 ± 0.1 (1.0–1.6)
**IND**	1.2 ± 0.1 (1–1.3)	1.1 ± 0.1 (1.0–1.4)	1.4 ± 0.2 (1.0–1.5)	1.1 ± 0.1 (1.0 –1.3)	1.2 ± 0.1 (1.0–1.3)	1.0 ± 0.1 (0.8–1.2)	0.9 ± 0.1 (0.8–1.2)
**UAL**	3.8 ± 0.2 (3.2–4.1)	3.6 ± 0.4 (2.9–4.1)	5.5 ± 0.6 (5.0–5.6)	3.9 ± 0.5 (2.8–3.9)	4.5 ± 0.3 (4.2–5.3)	3.5 ± 0.4 (2.9–4.2)	4.5 ± 0.4 (3.8–5.7)
**HAL**	2.8 ± 0.2 (2.6–3.0)	2.8 ± 0.6 (1.9–2.9)	3.4 ± 0.6 (2.8–4.2)	2.7 ± 0.3 (2.3–3.2)	3.2 ± 0.2 (2.8–3.3)	2.7 ± 0.2 (2.3–3.1)	3.6 ± 0.4 (2.5–4.5)
**THL**	6.8 ± 0.2 (6.4–7.2)	6.7 ± 0.3 (2.3–3.1)	8.7 ±1.4 (7.2–8.9)	6.5 ± 0.7 (5.4–7.2)	7.7 ± 0.6 (6.3–8.0)	6.2 ± 0.4 (5.1–6.3)	7.6 ± 0.7 (6.2–9.2)
**TAL**	6.7 ± 0.3 (6.3–7.1)	6.9 ±0.6 (4.2–7.3)	8.3 ± 1.0 (6.7–9.2)	5.7 ± 0.7 (4.8–7.0)	7.2 ± 0.6 (6.1–7.9)	5.8 ± 0.3 (5.1–6.3)	7.6 ± 0.7 (6.2–8.8)
**TL**	4.1 ± 0.2 (3.8–4.6)	4.6 ± 0.4 (4.3–6.3)	5.4 ± 1.4 (2.9–6.2)	3.8 ± 1.0 (4.2–7.0)	4.2 ± 0.6 (6.3–8.0)	3.8 ± 0.2 (3.6–4.3)	4.7 ± 0.8 (3.9–8.7)
**FL**	4.8 ± 0.4 (4.2–5.2)	5.2 ± 0.5 (4.7–6.1)	6.3 ± 1.3 (3.9–7.6)	4.4 ± 0.6 (3.2–5.4)	5.1 ± 0.4 (4.7–6.0)	4.3 ± 0.4 (5.5–3.0)	5.7 ± 0.6 (4.5–7.2)

**Table 2.** Individuals of the genus *Amazophrynella* (A) and *Dendrophryniscus* (D) used in the molecular analyses. Information includes samples, collecting locality, GenBank accession number for the 16S rDNA fragment, voucher number and specimen status.

Sample	Locality	Accession Number	Voucher number	Specimen status
*Amazophrynella javierbustamantei*	Quebrada Guacamayo, Peru	KR905184	MHNC 8331	Holotype
*Amazophrynella javierbustamantei*	Quebrada Guacamayo, Peru	KR905185	MHNC 8363	Paratype
*Amazophrynella matses*	Nuevo Salvador, Peru	KF681688	MZUNAP 928	Paratopotype
*Amazophrynella matses*	Nuevo Salvador, Peru	KF681689	MZUNAP 941	Paratopotype
*Amazophrynella minuta* sensu stricto	Taracuá, Brazil	KF792834	INPA-H 32729	Topotype
*Amazophrynella minuta* sensu stricto	Taracuá, Brazil	KF792835	INPA-H 32730	Topotype
*Amazophrynella amazonicola*	Puerto Almendras, Peru	KF681868	MZUNAP 901	Holotype
*Amazophrynella amazonicola*	Puerto Almendras, Peru	KF681669	MZUNAP 915	Paratopotype
*Amazophrynella vote*	Parque Nacional Nascentes do Lago Jari, Brazil	KF433970	INPA-H 28720	Paratype
*Amazophrynella vote*	Parque Nacional Nascentes do Lago Jari, Brazil	KF433971	INPA-H 28722	Paratype
*Amazophrynella bokermanni*	Juruti, Pará, Brazil	KF433975	INPA-H 31864	
*Amazophrynella bokermanni*	Juriti, Pará, Brazil	KF433976	INPA-H 31861	
*Amazophrynella manaos*	Mineração taboca, Brazil	KF433954	INPA-H 29566	Paratype
*Amazophrynella manaos*	Mineração taboca, Brazil	KF433957	INPA-H 29567	Paratype
Amazophrynella sp. aff. manaos	Mitaraka, French Guiana	JN867570	296MC	
Amazophrynella sp. aff. manaos	Mitaraka, French Guiana	EU201057	3035T	
Amazophrynella sp. aff. minuta	Rio Lagarto Cocha, Peru	AY326000	USNM 520905	
Amazophrynella sp. aff. minuta	Equador	DQ158262	QCAZ833	
*Dendrophryniscus proboscideus*	Mata Escura, Brazil	JN867566	MTR17173	
*Dendrophryniscus proboscideus*	Mata Escura, Brazil	JN867564	MTR17171	
*Dendrophryniscus oreites*	Serra das lontras, Brazil	JN867567	MTR16368	
*Dendrophryniscus carvalhoi*	Parna Caparão, Brazil	JN867568	MTR15755	
*Dendrophryniscus carvalhoi*	Parna Caparão, Brazil	JN867569	MTR15757	
*Dendrophryniscus leucomyxtas*	Ilha grande, Brazil	JN867558	MTR15547	
*Dendrophryniscus leucomyxtas*	Ilha grande, Brazil	JN867557	MTR15548	
*Dendrophryniscus berthalutzae*	Treviso, Brazil	JN867551	CFBH10322	
*Dendrophryniscus brevipollicatus*	Estação Biológica de Boracia, Brazil	JN867554	AF1541	
*Dendrophryniscus brevipollicatus*	Estação Biológica de Boracia, Brazil	JN867553	AF1175	

**Table 3.** Uncorrected *p*-distances between *Amazophrynella* (A), species and the sister genus *Dendrophryniscus* (D). Molecular distances are based on the 480-bp fragment of 16S rDNA. We included *Amazophrynella minuta* sensu stricto from its type locality and two candidate species, Amazophrynella sp. aff. manaos and Amazophrynella sp. aff. minuta mentioned in Fouquet et al. (2012a).

16S rDNA	1	2	3	4	5	6	7	8	9
1 *Amazophrynella amazonicola*									
2 *Amazophrynella matses*	0.08								
3 Amazophrynella sp. aff. minuta	0.06	0.07							
4 *Amazophrynella minuta*	0.05	0.08	0.03						
5 *Amazophrynella javierbustamantei* sp. n.	0.09	0.03	0.06	0.07					
6 *Amazophrynella vote*	0.12	0.12	0.12	0.12	0.13				
7 *Amazophrynella bokermanni*	0.12	0.12	0.11	0.11	0.13	0.10			
8 *Amazophrynella manaos*	0.12	0.12	0.12	0.14	0.12	0.10	0.08		
9 Amazophrynella sp. aff. manaos	0.12	0.11	0.12	0.13	0.12	0.10	0.07	0.04	
10 *Dendrophryniscus leucomystax*	0.19	0.21	0.17	0.18	0.20	0.22	0.18	0.20	0.20

**Table 4.** Species level diagnostic characters observed in the 16S rDNA gene of *Amazophrynella javierbustamantei* sp. n. and other species of genus *Amazophrynella*. First line indicates position of the character within the 16S rDNA gene; (-) indicates a deletion.

Species	213	232	271	276	470	471	473	474	476	477	478	479	480
*Amazophrynella manaos*	A	C	A	C	A	T	G	T	C	A	A	A	A
*Amazophrynella vote*	A	T	A	C	C	C	C	T	T	A	A	A	G
*Amazophrynella minuta*	C	T	A	A	C	C	C	T	T	A	A	A	G
*Amazophrynella bokermanni*	A	T	A	C	A	T	G	T	C	A	A	A	A
*Amazophrynella amazonicola*	C	C	A	C	C	C	C	T	T	A	A	T	G
*Amazophrynella javierbustamantei* sp. n.	T	G	G	T	T	G	T	G	A	G	C	C	-
*Amazophrynella matses*	C	T	A	C	C	C	C	T	T	A	A	T	T

**Table 5.** Character loadings, eigenvalues, and percentage of explained variance for Principal Components (PC) 1–2. The analysis was based on eleven morphometric variables of adult males: *Amazophrynella minuta* complex (*Amazophrynella minuta* sensu stricto; *Amazophrynella amazonicola*; *Amazophrynella matses* and *Amazophrynella javierbustamantei* sp. n.).

Variables	PC1	PC2
HW	0.462	-0.146
HL	0.455	-0.104
SL	0.374	-0.244
ED	0.261	0.052
IND	0.369	-0.271
UAL	0.139	0.258
HAL	-0.032	0.484
THL	0.311	-0.295
TAL	0.314	0.350
TL	0.116	0.364
FL	0.063	0.433
% of variation	24.93	23.63
%	24.93	48.56

**Figure 3.**
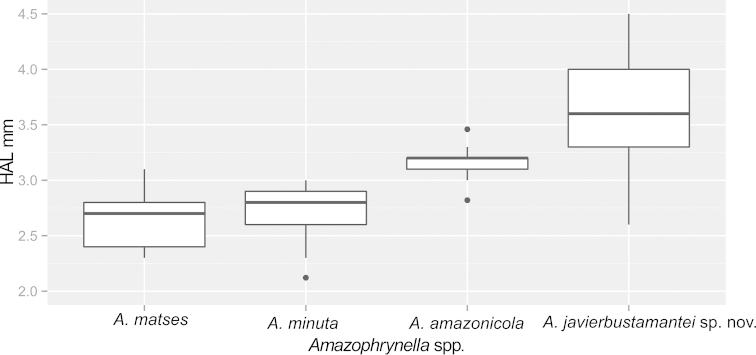
Measurement comparison of the Hand Length (HAL) between species of *Amazophrynella minuta* complex.

All the species of the group are significantly different in shape (MANOVA, *F*_24.3_, *Pillae´s trace* < 0.001). The discriminate function analysis (DFA) found specimens correctly classified in 56.6% of cases and a moderate prior probabilities of groups (*Amazophrynella minuta* = 28.75%, *Amazophrynella amazonicola* = 18.75%, *Amazophrynella matses* = 16.25% and *Amazophrynella javierbustamantei* sp. n. = 36.25%). The variables that contributed most to the classification were HAL, SVL and TAL (Table 6). The differences in HAL were significant (ANOVA, *F*_45.27_, *P* < 0.001) among all the species of *Amazophrynella minuta* “species complex” (see Fig. 1), and reveals *Amazophrynella javierbustamantei* sp. n. as the species with the largest HAL (Fig. 3).

**Table 6.** Character loadings of explained variance for Discriminant Function Analysis (DFA). The analysis was based on twelve morphometric variables of adult males of the *Amazophrynella minuta* complex (*Amazophrynella minuta* sensu stricto; *Amazophrynella amazonicola*; *Amazophrynella matses* and *Amazophrynella javierbustamantei* sp. n.).

Variables	Discriminant Function
SVL	6.343
HW	-7.628
HL	0.146
SL	-5.479
ED	-1.175
IND	-6.015
UAL	1.313
HAL	5.744
THL	-3.871
TAL	13.944
TL	-1.250
FL	1.016

### Morphological description

#### Amazophrynella javierbustamantei sp. n.

Taxon classificationAnimaliaAnuraBufonidae

http://zoobank.org/A946B949-1D1F-4FF5-B722-0B33435EE610

##### Holotype

**Figure 4.**
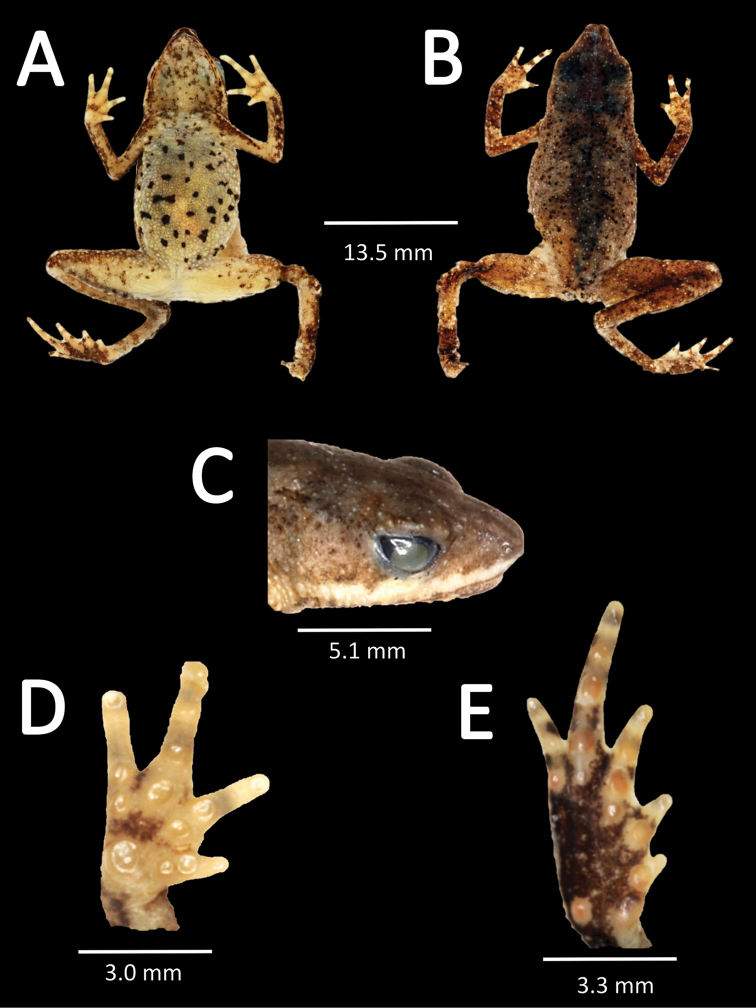
Holotype of *Amazophrynella javierbustamantei* sp. n. (MHNC 8331); **A** dorsal view **B** ventral view **C** dorsolateral view **D** right hand **E** right foot.

(Fig. 4). MHNC 8331 (Genbank *16S rRNA*: KR905184). Adult male, collected at Quebrada Guacamayo (12°54'24.5"S; 69°59'32.7"W, 215 m a.s.l.) km 105 of the highway Puerto Maldonado–Cusco City, District Inambari, Province Tambopata, Department Madre de Dios, Peru, on 27 October 2009 by Juan C. Chaparro and Oscar Quispe.

##### Paratypes

**Figure 5.**
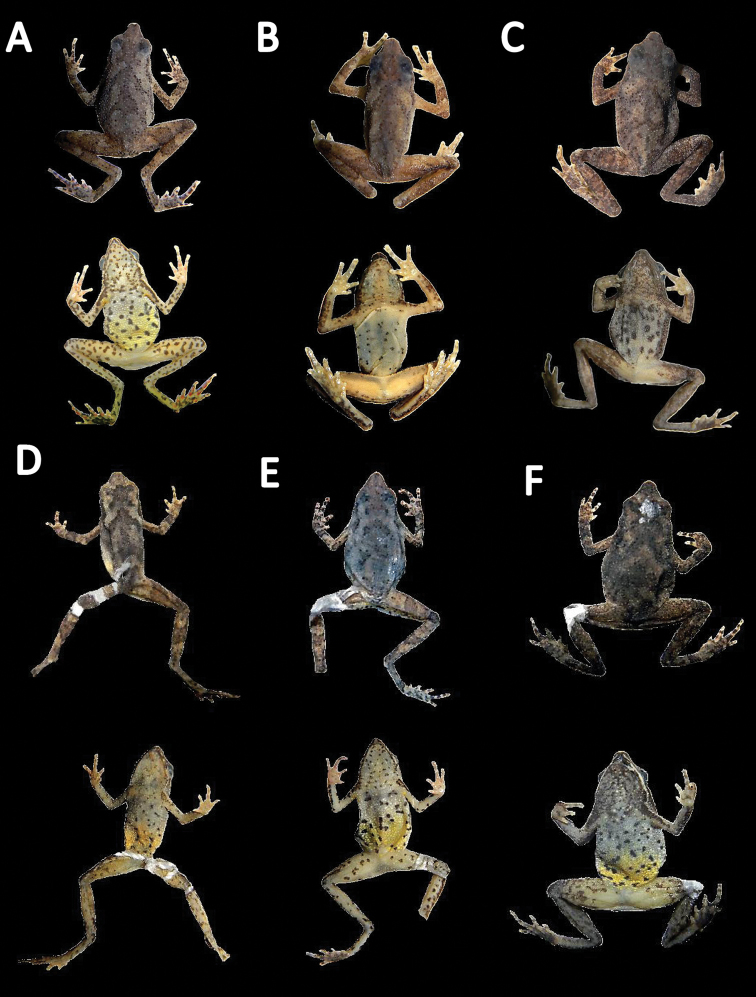
Dorsal and ventral view of some Paratypes of *Amazophrynella javierbustamantei* sp. n. Adult males (MHNC 8245: SVL 13.6 mm; MHNSM 31255: SVL 15.9 mm; MHNSM: 17993 SVL 14.2 mm; Adult females (MHNC 11002: SVL 17.2 mm, MHNC 9739: SVL 21.5, MHNC 8362: SVL 18.0 mm).

(Fig. 5). Twenty-two specimens (males= 09, females= 13). MHNC 8363, MHNC 8245, MHNC 8238, adult males, MHNC 8316, MHNC 8484, MHNC 8362, MHNC 8354, adult females, collected with the holotype (12°28'25"S, 69°12'36"W, 205 m a.s.l.). MHNC 11001, adult male, MHNC 11002, MHNC 11003, MHNC 11004, adult females collected by E. Aguilar on 17 May 2009, from La Pampa km 107 highway Puerto Maldonado–Cusco City, Department Madre de Dios (12°40'14.14"S, 72°27'30"W, 250 m a.s.l.). MHNSM 17993, adult male collected by A. Angulo in 1999; from Province Manu, locality of Inambari, Department Madre de Dios (13°02'29.28"S, 70°22'46.65"W, 306 m a.s.l.). MHNSM 25651, adult female, collected by D. Rodriguez on April 2007, from Province La Convención, locality of Camana, Department Cusco (12°05'9.25”S, 73°03'2.61”W, 680 m a.s.l.). MHNC 9939, MHNC 9940, adult females, collected by J. Delgado on 17 January 2010 from Province La Convención, locality of Mapi, Department Cusco (11°31'19.17”S, 73°28'29.83”W, 708 m a.s.l.). MHNC 9387, adult male, collected by G. Estrada on 21 January 2010, from locality of Tambo Poyeni near Quebrada Mayapo, Department Junin (11°19'29.9”S, 73°32'16.7"W, 388 m a.s.l.). MHNC 9754, MHNC 9756, adult males, MHNC 9626 , MHNC 9679, MHNC 9680, MHNC 9757, adult females, collected by A. Pari on January 2010, from locality of Tsoroja, Department Junin (11°18'56.06”S, 73°32'32.11”W, 399 m a.s.l. and 11°23'14.50”S , 73°29'43.00”W, 450 m a.s.l.).

##### Diagnosis.

The new species is part of *Amazophrynella* based on molecular phylogenetic relationships (Fig. 1) and morphological synapomorphies (Fouquet et al. 2012a).

*Amazophrynella javierbustamantei* sp. n. is characterized by: (1) skin on dorsum tuberculate, with many subconical tubercles disperse on arms, legs, head and body; ventral skin coarsely areolate, throat and chest aerolate; (2) tympanic membrane and tympanic annulus not apparent through the skin; (3) snout long, subacuminated, protruding in lateral views; (4) upper eyelid with smaller tubercles, cranial crests absent; (5) dentigerous process of vomers absent; (6) vocal sac, vocal slits and nuptial pads absent; (7) finger I shorter than finger II, tips of digits rounded; (8) fingers lacking lateral fringes; (9) ulnar tubercles present; (10) heel bearing eight or more small low tubercles, tarsus with small tubercles and lack of folds; (11) plantar surfaces of feet bearing one metatarsal tubercle, the inner 2.5x larger than the outer, outer subconical; supernumerary plantar tubercles round and low; (12) toes lacking lateral fringes; webbing basal; toe III equal than toe V, tips of digits rounded; (13) dorsally is dark brown to light brown, and gray to black in some, ventrally, cream with yellow to orange marks, with black to dark brown spots; (14) SVL 16.39–22.25 mm in females, 12.79–16.42mm in males; (15) hand length is the greatest of all species of *Amazophrynella*: 3.6 mm in males (n= 26) and 4.6 mm in females (n=20), see Fig. 3; (16) thirteen molecular autapomorphies in the 16S rDNA gene.

##### Comparison with other species.

**Figure 6.**
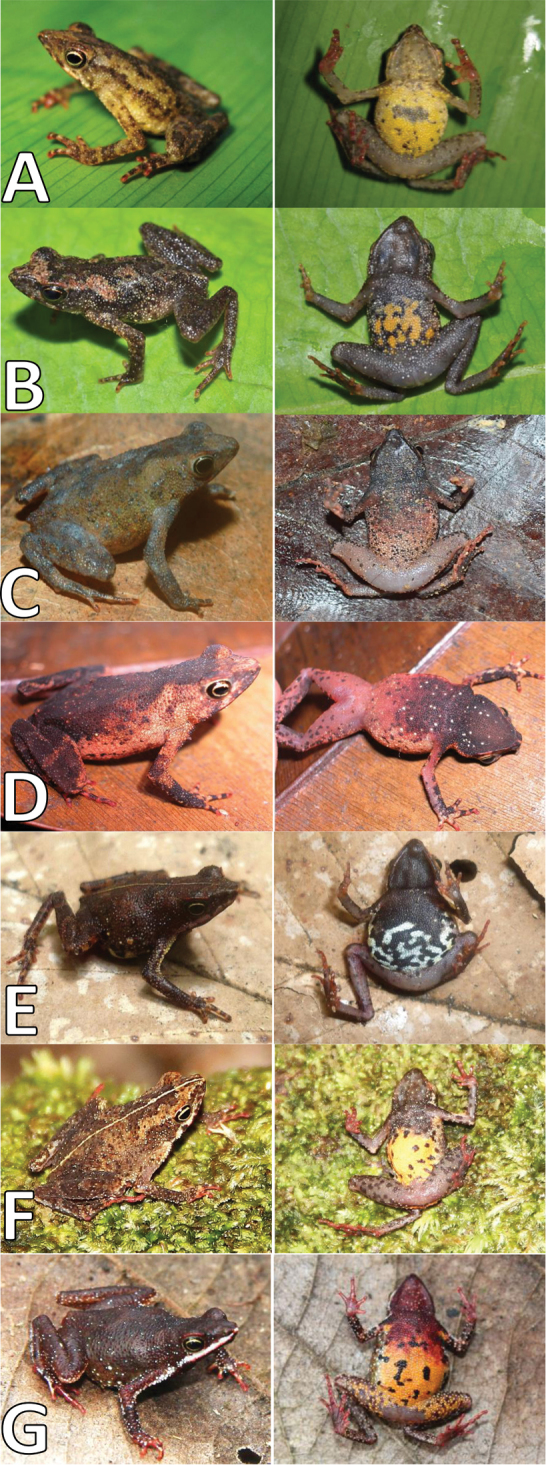
Dorsal and ventral morphological comparison between the *Amazophrynella* spp. (Unvoucher specimens): **A** *Amazophrynella javierbustamantei* sp. n. **B** *Amazophrynella minuta* **C** *Amazophrynella bokermanni* **D** *Amazophrynella vote* **E** *Amazophrynella manaos* **F** *Amazophrynella matses* **G** *Amazophrynella amazonicola*.

*Amazophrynella javierbustamantei* sp. n. (Figs 4, 5, 6) differs in the following character states (states of other species in parentheses). From *Amazophrynella minuta* (Fig. 6A) by having body skin texture tuberculate (roughly granular); relative abundance of spiny granules on the forelimbs (prickly warty skin on axillary region of the forelimbs); absence of large warts on dorsum (presence of large warts); throat and chest cream-grayish (light brown); posterior side of belly color pale orange yellowish with tiny rounded black or dark brown spots (throat and the whole belly intensely orange yellowish); tiny rounded black spots covering the belly (irregular black ocelli or blotches); metatarsal tubercle rounded (oval). From *Amazophrynella bokermanni* (Fig. 6B) relative size of fingers, with finger I shorter than II (I>II); snout vent length smaller in males (15.8 mm) and females (22.25 mm) (*Amazophrynella bokermanni* with maximum 22 mm SVL in males and 28 mm SVL in females, see Izecksohn 1993); smaller snout in males, with 2.2 mm SL, n = 26 (2.7 mm SL, n = 5; see Table 1); posterior side of belly color pale orange yellowish with tiny rounded black or dark brown spots (white coloration with small black dots). From to *Amazophrynella vote* (Fig. 6C) snout subacuminated in dorsal view (rounded); posterior side of belly color pale orange yellowish with tiny rounded black or dark brown spots (ventral color pattern reddish brown, with presence of small white dots). From *Amazophrynella manaos* (Fig. 6D) snout subacuminated (snout truncate); dorsal skin finely granular (dorsal surfaces granular); throat and chest grayish (dark coloration); posterior side of belly color pale orange yellowish with tiny rounded black spots (venter cream with black spots or stripes). From to *Amazophrynella matses* (Fig. 6E) snout subacuminated (snout slightly truncate), edges of nasal protrusion not dilated (dilated in ventral view); shape of palmar tubercle rounded (palmar tubercles elliptical); finger tips unexpanded (expanded), rounded tiny black spots covering the belly (medium-sized black ocelli or streaks); coloration of the belly pale yellow (belly completely yellow). From *Amazophrynella amazonicola* (Fig. 6F) by the absence of small triangular protrusion on the tip of the snout in both dorsal and ventral views (presence); body surface granular (finely granular), dorsum uncovered with medium-sized granules scattered irregularly (covered with medium-sized granules scattered irregularly); posterior side of belly color pale orange yellowish with tiny rounded black or dark brown spots (orange yellowish with dark red and brown blotches).

##### Description of the holotype.

Body slender, head triangular, slightly longer than wide; head length 35.5% of SVL, head width 30.9% of SVL. Snout long, subacuminate in dorsal view, protruding in lateral view; *canthus rostralis* straight and loreal region vertical; without papilla; snout length 39.0% of head length; tympanic membrane and tympanic annulus not apparent through the skin, skin of the tympanic area covered by round sub-conical warts; vocal sac externally not visible, vocal slits absent; eyes prominent 23.8% of head length; upper eyelid covered with small tubercles; those close to the external margin aligned in a more or less distinct row; nostril closer to snout than to eyes; internarial distance smaller than eye diameter; presence of a line of small spiny granules from the outer edge of the mouth to upper arm, choanas small and circular.

Dorsal skin finely tuberculate with several large tubercles scattered sub-conical tubercles on upper arm; texture of ventral skin granular, covered by rounded granules. Dorsolateral surfaces, granular, with presence of large rounded tubercles. Forelimbs slender, upper arm length 29.6% of SVL; edges of lower arm and upper arm finely tuberculate with several large sub-conical and spiny granules; hand length 76.5% of upper arm length; fingers slender, tips not expanded; relative length of fingers I<II<IV<III; supernumerary tubercles and accessory palmar tubercles present, palmar large and rounded, supernumerary tubercles low, small rounded; subarticular tubercles rounded and small, one tubercle on fingers I, II and IV and two on finger III; fingers I and II basally webbed; indistinct nuptial pad. Hind limbs slender; ventral skin from thigh to tarsus covered by spiny tubercles, foot length 66% of thigh length; relative length of toes I<II<V<III<IV: inner metatarsal tubercle oval, 2.5× larger than outer; outer metatarsal tubercles small, rounded; subarticular tubercles present, rounded, present one on fingers I, II, and two on fingers III, V and three on finger IV; and tip of toes not expanded.

##### Measurements of the holotype

(in millimeters). SVL 15.1; HW 4.6; HL 5.3; SL 2.1; ED 1.2; IND 1.0; UAL 4.4; HAL 3.4; THL 8.1; TAL 8.1; TL 4.5; FL 5.3.

##### Coloration of the holotype.

In life: dorsum of the holotype mostly light brown with dark brown in the dorsum; dorsolaterally creamish-brown with scattered black blotches; dorsal surfaces of hands and feet creamish-brown, and gray on arms and legs; belly creamish-gray with black dots, and the throat gray; fingers, toes and plantar surfaces reddish-black; groin with orange marks; iris with a bronze ring; cloaca with orange flap, black pupil and bronze iris. In alcohol: dorsum brownish-grey; venter cream with black and brown dots; orange surfaces turned cream, with a white longitudinal stripe on upper jaw extending from nostril to forearm.

##### Variation.

**Figure 7.**
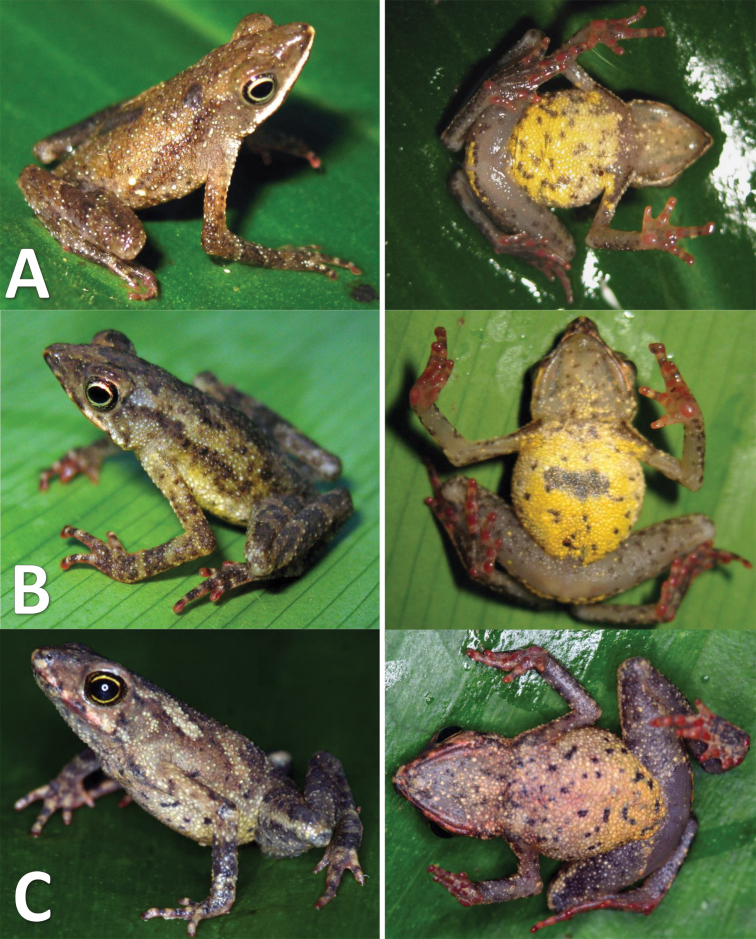
Dorsal and ventral variation of *Amazophrynella javierbustamantei* sp. n. (Unvoucher specimens): **A–C** Nueva Arequipa, Madre de Dios Department **B** Basin of Bajo Urubamba, Cusco Department.

The new species is phenotypically variable. In some individuals (e.g. MHNC 8245 and MHNC 11002, see Fig. 5) patterning on the dorsum varies, with these specimens presenting brown chevrons extending from the head to the vent. Some individuals showed a white line extending from the tip of the nose to the upper arm. Another specimen (MHNC 9739, see Fig. 5) presented a yellow pale coloration in the axillary region (in ventral view). In some individuals, the coloration of the throat extended onto the chest (e.g. MHNC 11002, MHNC 9739 and MHNC 8245, see Fig. 5). The pale yellow coloration of the belly surface may extend from thighs to the chest or just to the middle of the belly (e.g. MHNC 8362, see Fig. 5 and Fig. 7B). In some individuals, the thighs are abundantly covered by rounded tiny spots extending to the shank (Fig. 7B). In preserved specimens the dorsum becomes light brown and the belly coloration vary from white to yellow pale (e.g. MHNSM 31255 and MHNSM 17993, see Fig. 5). The color of the finger becomes pale red and in other individuals the red coloration of the fingers became brown or orange (Fig. 5).

##### Bioacoustics.

**Figure 8.**
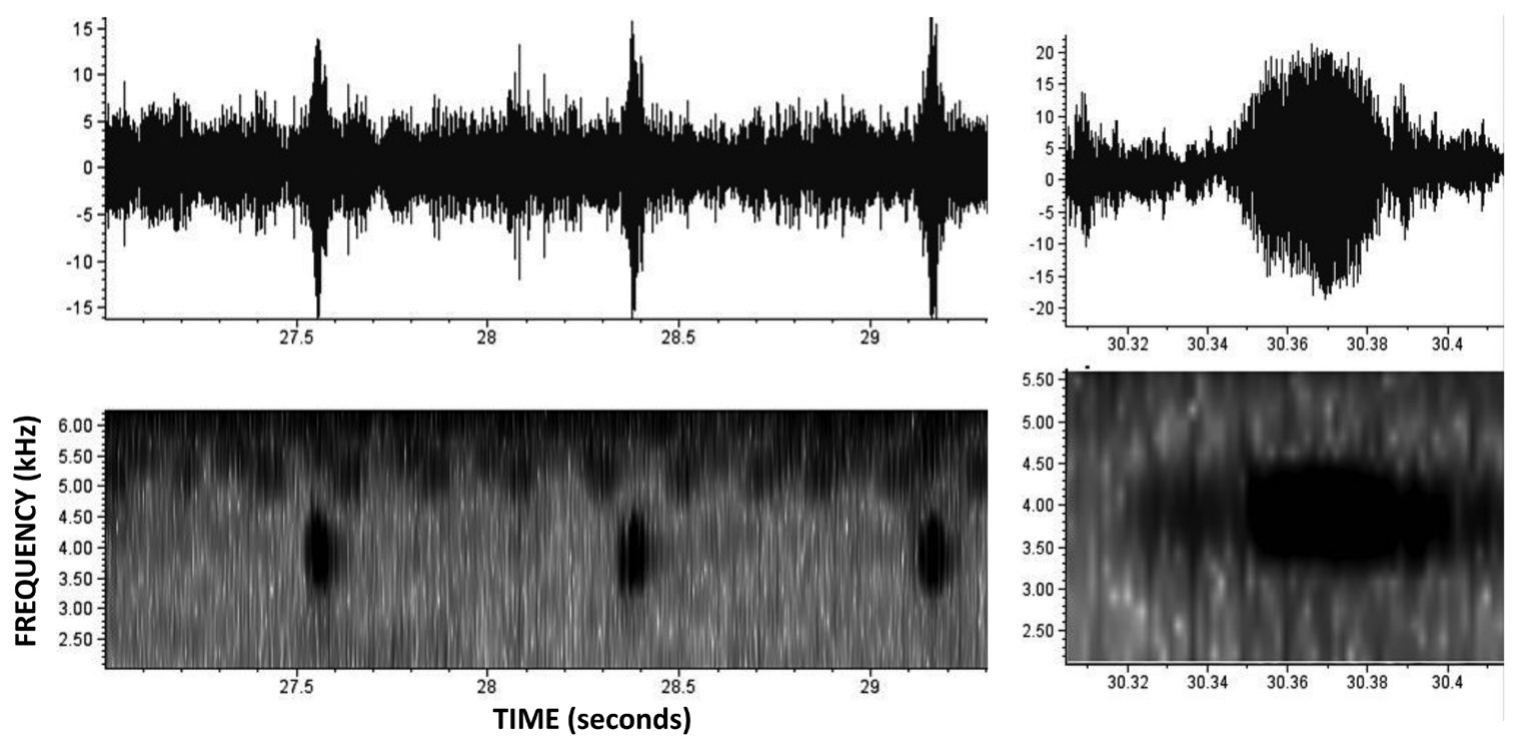
Advisement call of *Amazophrynella javierbustamantei* sp. n. from the Tambopata National Reserve, Madre de Dios, Peru (207 meters a.s.l.) (Macauly Library of Natural Songs and Cornell Laboratory of Ornithology) by the authors Crocoft, Morales and Mc Diarmid (2007). **A** Oscilogram and spectrogram by one note **B** Oscilogram and spectrogram of notes from the advisement Call.

The following values are presented as: min-max (average ± SD, number of notes). The call is a trill type call issued during continuous and regular intervals (Fig. 8). Each note had a duration of between 0.03 to 0.08 seconds (0.05 ± 0.01 seconds, n = 20). The number of pulses varied between 8 to 18 pulses per note (10.4 ± 2.6 pulses/note, n = 20). The silence between notes varied from 0.4 to 1.6 seconds (0.8 ± 0.3 seconds, n = 20). The dominant frequency varied from 3962.1 to 3789.8 kHz (3927.6 ± 70.7 kHz, n = 20), and coincides with the fundamental frequency. Time to peak amplitude was around 0.014 to 0.04 seconds (0.02 ± 0.01 seconds, n = 20).

##### Distribution, ecology and conservation.

**Figure 9.**
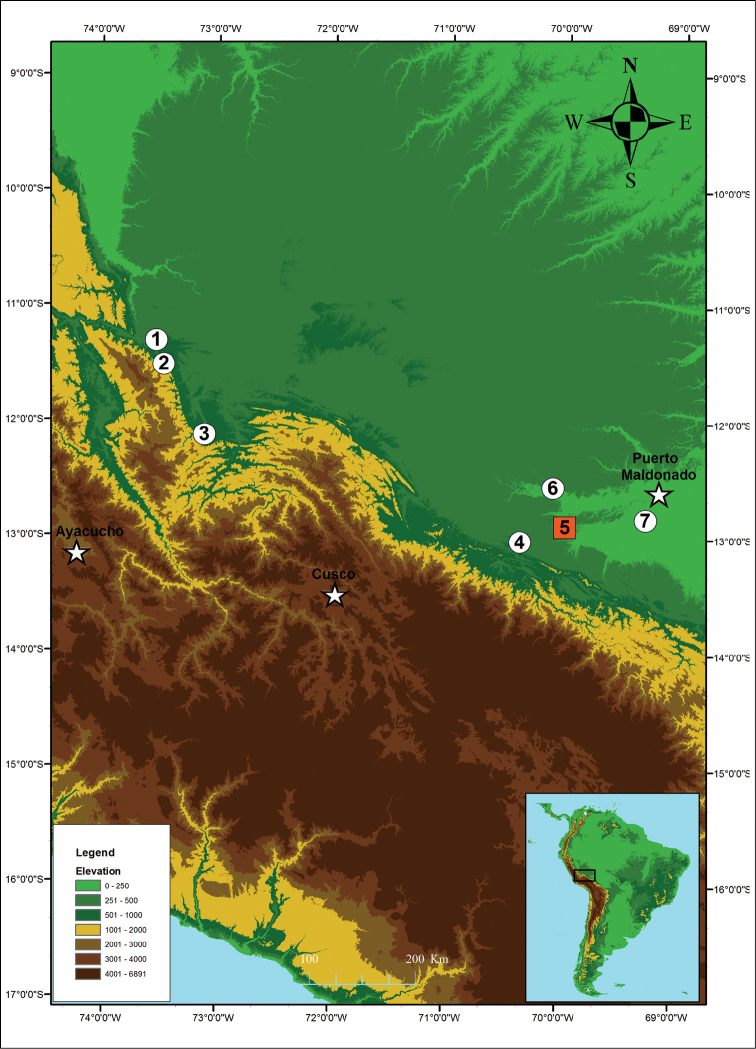
Distribution map of *Amazophrynella javierbustamantei* sp. n. in Peru. Holotype locality in square orange, **5** Guacamayo Creek, Department Madre de Dios. Paratypes localities in white circles **1** Tsoroja, Department Junin **2** Mapi, Department Cusco **3** Camana, Department Cusco **4** Inambari, Department Madre de Dios **6** Los Amigos Biological Station, Department Madre de Dios **7** Explorer’s Inn, Department Madre de Dios.

*Amazophrynella javierbustamantei* sp. n. is known from the Department of Cusco, in the lower Urubamba river basin and Department of Madre de Dios (Inambari, Candamo and Nueva Arequipa) in Peru (Fig. 9). Its distribution can vary from 215 m a.s.l. to 708 m a.s.l. Additional specimens were recorded at Los Amigos Biological Station , Tapir Lodge, and Explorers Inn, in Tambopata National Reserve. Individuals were active during the day, jumping on leaf litter, at night they were sleeping on leaves around 30 cm above ground. This species breeds close to the edges of permanent oxbow lakes, males call during the day while perched above streams in tangles (Cocroft et al. 2001). Three of the localities, km 105, 107 and 117 of the highway Puerto Maldonado–Cusco, Department Madre de Dios, show evidence of serious environmental impacts due to illegal gold mining activities, with forest and soil removed, and environmental pollution via organic and inorganic chemicals and heavy metal (specially mercury) poisoning. In addition, the new species is distributed inside of territories where oil companies are operating. On the other hand, the species is present in two protected areas, the Tambopata Natural Reserve and Machiguenga Communal Reserve. The conservation status of this species remains unknow, but was listed in 2008 as Least Concern on the IUCN red list (2015), because it was confused with *Amazophrynella minuta*, and because *Amazophrynella minuta* s.l. had wide distribution at that time, apparent tolerance of a certain degree of habitat modification, presumed large population, and because it is unlikely to be declining, and thus did not qualify for listing in a more threatened category. With recent studies the genus, the species complex of *Amazophrynella minuta*, was split in five species, three of them are now formally described for Peru (*Amazophrynella matses*, *Amazophrynella amazonicola* and *Amazophrynella javierbustamantei* sp. n.). The recognition of these new species will require the reevaluation of the conservation status of these species. It should also act as an impetus for additional field and laboratory studies of Peruvian amphibians, in order to understand the real conservation status of this fauna.

##### Etymology.

The species is named after Dr. Javier Bustamante, a Peruvian residing in United States, to whom we dedicate this species in recognition of his friendship and support of herpetological taxonomy and systematics research and amphibian conservation in Peru.

## Discussion

Taxonomic reviews of Amazonian amphibians suggests that morphological characters are too conservative to permit delimiting species since closely related species share similar morphologies, and amphibians in general are morphologically conservative (e.g., Elmer et al. 2007; Fouquet et al. 2007c; Funk et al. 2011; Padial et al. 2009). Thus, the use of integrative techniques in taxonomy is revolutionizing the identification and delimitation of species based on independent lines of evolutionary evidence (Dayrat 2005; Padial and De la Riva 2009). The use of an integrative approach not only allows for the discovery and delimitation of new species, it also helps us to understand the mechanism of species formation. Thus, integrative taxonomy allows us to have a better understanding of the true scope of anuran diversity in the Amazon, and it allows us to better understand the processes that generated this biodiversity.

The taxonomic ambiguity surrounding the name *Amazophrynella minuta* and to a lesser extent *Amazophrynella bokermanni* resulted in a severe underestimation of the taxonomic diversity of this genus. Since the descriptions of *Amazophrynella minuta* in 1941 and *Amazophrynella bokermanni* in 1993, the taxonomy of the genus has not been revised, leading to misdiagnoses of other species as either *Amazophrynella minuta* or *Amazophrynella bokermanni* due to the relatively generalized descriptions of these taxa. Three publications since 2012 (Ávila et al. 2012; Rojas et al. 2014, 2015) described four new species, increasing the taxonomic diversity of the genus by 200%. All four species were previously classified as populations of a single species with a large distribution (*Amazophrynella minuta* sensu lato). Although striking, the severe underestimation of taxonomic diversity observed in *Amazophrynella* and the existence of multiple lineages in *Amazophrynella minuta* is nothing particular to this group. Examples of other Amazonian species complexes include *Rhinella margaritifera* and *Scinax ruber*, *Pristimantis ockendeni*, *Pristimantis fenestratus*, *Engystomops petersi*, *Hybsiboas fasciatus*, *Dendropsophus minutus* and *Osteocephalus taurinus* (Fouquet et al. 2007; Elmer and Canatella 2008; Padial et al. 2009; Funk et al. 2011; Caminer and Ron 2014; Gehara et al. 2014, Jungfer et al. 2013).

The descriptions by Rojas et al. (2014, 2015) were based, in part, on diagnostic characters observed in the 16S rDNA. This gene is widely used as a DNA barcode for amphibians, for reliable species identification (Vences et al. 2005, Fouquet et al. 2007), for evaluating monophyly of species and for discovering divergent lineages (Padial et al. 2009, Crawford et al. 2010; Padial et al. 2010 and Padial et al. 2012). Based on 16S rDNA analyses, we also have evidence that *Amazophrynella bokermanni* and *Amazophrynella vote* represent species complexes (RRRZ, personal observation). This observation is in addition to the existence of the two candidate species of *Amazophrynella* already observed in previous analyses: one from the Guiana Shield (Amazophrynella sp. aff. manaos), sister taxon of *Amazophrynella manaos*, and another from Ecuador (Amazophrynella sp. aff. minuta), sister taxon of *Amazophrynella minuta* sensu stricto (Fig. 1). Although the taxonomic status of these candidate species will need to be confirmed using morphological and bioacoustics data, it is clear that even with the recent descriptions, the taxonomic diversity of the genus remains underestimated.

While part of our evidence for the existence of the new species as well as those described previously by Rojas et al. (2014, 2015) comes from the use of molecular data, the descriptions make use of other data types and non-molecular diagnoses. Thus these undiscovered lineages were not truly cryptic (morphologically cryptic), but rather the result of poor taxonomic knowledge of the group. In this respect, the genus *Amazophrynella* again is not the exception, but rather the norm.

The new species *Amazophrynella javierbustamantei* sp. n. is clearly differentiated in multivariate morphometric space from the other members of the *Amazophrynella minuta* “species group” (*Amazophrynella minuta*, *Amazophrynella amazonicola* and *Amazophrynella matses*). Together with the description of *Amazophrynella javierbustamantei* sp. n. we also provide advertisement call. *Amazophrynella javierbustamantei* sp. n. is only the second species of the genus for which an advertisement call is known and recorded (see Duellman 1978). Acoustics can provide evidence of potentially new species with behavioral or premating isolating mechanisms (e.g. De la Riva et al. 1997; Gerhardt 1998; Simões et al. 2008, Padial and De la Riva 2009; Padial et al. 2012), thus providing evidence of evolutionary mechanisms that contributed to the species diversity of the genus *Amazophrynella*.

The threats to the biological conservation of *Amazophrynella javierbustamantei* sp. n. are evident, with uncontrolled exploration for gold, illegal mining and the destruction of habitat in the Departments of Madre de Dios and Cusco, probably causing a significant reduction in the population sizes of the species and fragmenting its distribution. For these reasons is necessary to analyze the current population status and trends of this and another amphibian species in this Department of southern Peru.

## Supplementary Material

XML Treatment for Amazophrynella javierbustamantei

## Appendix 1

### Specimens examined

*Amazophrynella minuta*—BRAZIL: Taracuá, Uaupés River: INPA-H 32725, INPA-H 32723, INPA-H 32729, INPA-H32730, INPA-H32736, INPA-H32731 (females) and INPA-H 32724, INPA-H32728, INPA-H 32733, INPA-H 32735, INPA-H 32722, INPA-H 32738, INPA-H 32737, INPA-H 32739. INPA-H 32720, INPA-H 32732, INPA-H 32726, INPA-H 32730, INPA-H 32740, INPA-H 32734, INPA-H 32721 (males).

*Amazophrynella bokermanni*—BRAZIL: Juriti, Pará: INPA-H 31861, INPA-H 31864, INPA-H 31863, INPA-H 31862, INPA-H 31865, municipality of Juriti, Pará State, Brazil (50 km from type locality).

*Amazophrynella vote*—BRAZIL: Fazenda São Nicolau, Cotriguaçu, Mato Grosso (Holotype: UFMT-A 11138); Madeira River, Manicoré (Paratypes: INPA-H 12256, 12331, 12255, 12342, 12343, 12366, 12267); Aripuanã River, Novo Aripuanã (Paratype: INPA-H 12326); Parque Estadual do Guariba: Manicoré (Paratypes: INPA-H 21558); Parque Nacional Nascentes do Lago Jari, Tapauá: Amazonas (Paratypes: INPA-H 27412, 27417-27419, 27421-27423, 27425-27426).

*Amazophrynella manaos*—BRAZIL: Campus da Universidade Federal do Amazonas, Amazonas (Holotype: INPA-H 31866, paratypes: INPA-H 6983, INPA-H 6984, INPA-H 6987, INPA-H 7797); Presidente Figueiredo, Amazonas (Paratypes: INPA-H 29568, INPA-H 29569, INPA-H 29571, INPA-H 29570, INPA-H 29572, INPA-H 20986; INPA-H 21217, INPA-H 30577, INPA-H 30575, INPA-H 30573, INPA-H 30572, INPA-H 30576); Reserva Florestal Adolpho Ducke, Amazonas (INPA-H 21028, INPA-H 21170, INPA-H 21060, INPA-H 31866, INPA-H 21007, INPA-H 21008, INPA-H 21009, INPA-H 21010, INPA-H 21011, INPA-H 21012, INPA-H 21013).

*Amazophrynella amazonicola*—PERU: Puerto Almendra San Juan Bautista, Loreto (Holotype: MZUNAP 901, paratopotypes: MZUNAP 906; MZUNAP 915; MZUNAP 110; MZUNAP 907, MZUNAP 917; MZUNAP 889; MZUNAP 910; MZUNAP 911; MZUNAP 916; MZUNAP 913; MZUNAP 914; paratypes: MZUNAP 906; MZUNAP 915; MZUNAP 110; MZUNAP 907, MZUNAP 917; MZUNAP 889; MZUNAP 910; MZUNAP 911; MZUNAP 916; MZUNAP 913; MZUNAP 914); 58 km of Iquitos–Nauta highway on Fundo Zamora, San Juan Bautista, Loreto (Paratypes: MZUNAP 908, MZUNAP 924, MZUNAP 886, MZUNAP 900, MZUNAP 888, MZUNAP 919, MZUNAP 902, MZUNAP 887, MZUNAP 905, MZUNAP 920); Nauta, Maynas (Paratypes: MZUNAP 918, MZUNAP 909); Fundo UNAP, Maynas, Loreto (Paratype: MZUNAP 242)

*Amazophrynella matses*—PERU: Nuevo Salvador, Requena, Loreto (Holotype: MZUNAP 921, paratopotypes: MZUNAP 934, MZUNAP 955 MZUNAP 940, MZUNAP 948 MZUNAP 943, MZUNAP 952, MZUNAP 953, MZUNAP 958, MZUNAP 922, MZUNAP 923, MZUNAP 925, MZUNAP 926, MZUNAP 927, MZUNAP 944, MZUNAP 938, MZUNAP 936); Jenaro Herrera, Requena, Loreto (Paratypes: MZUNAP 928, MZUNAP 929, MZUNAP 930, MZUNAP 931, MZUNAP 933, MZUNAP 955, MZUNAP 935, MZUNAP 950, MZUNAP 937, MZUNAP 939, MZUNAP 941, MZUNAP 942, MZUNAP 946, MZUNAP 947, MZUNAP 949).

*Amazophrynella javierbustamantei* sp. n. —PERU: Quebrada Guacamayo, Tambopata, Madre de Dios (Holotype: MHNC 8331; MHNC 8363, MHNC 8245, MHNC 8238, MHNC 8316, MHNC 8484, MHNC 8362); La Pampa, Tambopata, Madre de Dios (MHNC 11101, MHNC 11102, MHNC 11103, MHNC 11104); Inambari, Manu, Madre de Dios (MHNSM 17993); Nuevo Arequipa, Tambopata, Madre de Dios (MHNC 8363, MHNC 8245, MHNC 8331, MHNC 8238, MHNC 8354, MHNC 8316, MHNC 8484, MHNC 8362); Rio Tambopata, Tambopata, Madre de Dios (MHNSM 9635, MHNSM 9641, MHNSM 9647, MHNSM 9642, MHNSM 9648, MHNSM 9633, MHNSM 9644, MHNSM 9646, MHNSM 9657, MHNSM 9640); Camana, La Convencion, Madre de Dios (MHNSM 25651); Mapi, La Convencion, Madre de Dios (MHNC 9939, MHNC 9940); Tambo Poyeni, Junin (MHNC 9387); Tsoroja, Junin (MHNC 9754, MHNC 9756, MHNC 9626, MHNC 9679, MHNC 9680, MHNC 9757). Rio Urubamba, Urubamba, Cusco (MHNC 9939, MHNC 9626, MHNC 9686, MHNC 9679, MHNC 9940, MHNC 9757, MHNC 9387, MHNC 9754, MHNC 9756).
